# Knowledge-Based Analysis for Detecting Key Signaling Events from Time-Series Phosphoproteomics Data

**DOI:** 10.1371/journal.pcbi.1004403

**Published:** 2015-08-07

**Authors:** Pengyi Yang, Xiaofeng Zheng, Vivek Jayaswal, Guang Hu, Jean Yee Hwa Yang, Raja Jothi

**Affiliations:** 1 Epigenetics & Stem Cell Biology Laboratory, National Institute of Environmental Health Sciences, National Institutes of Health, Research Triangle Park, Durham, North Carolina, United States of America; 2 Biostatistics Branch, National Institute of Environmental Health Sciences, National Institutes of Health, Research Triangle Park, Durham, North Carolina, United States of America; 3 Centre for Mathematical Biology, School of Mathematics and Statistics, University of Sydney, Sydney, Australia; University of California San Diego, UNITED STATES

## Abstract

Cell signaling underlies transcription/epigenetic control of a vast majority of cell-fate decisions. A key goal in cell signaling studies is to identify the set of kinases that underlie key signaling events. In a typical phosphoproteomics study, phosphorylation sites (substrates) of active kinases are quantified proteome-wide. By analyzing the activities of phosphorylation sites over a time-course, the temporal dynamics of signaling cascades can be elucidated. Since many substrates of a given kinase have similar temporal kinetics, clustering phosphorylation sites into distinctive clusters can facilitate identification of their respective kinases. Here we present a knowledge-based CLUster Evaluation (CLUE) approach for identifying the most informative partitioning of a given temporal phosphoproteomics data. Our approach utilizes prior knowledge, annotated kinase-substrate relationships mined from literature and curated databases, to first generate biologically meaningful partitioning of the phosphorylation sites and then determine key kinases associated with each cluster. We demonstrate the utility of the proposed approach on two time-series phosphoproteomics datasets and identify key kinases associated with human embryonic stem cell differentiation and insulin signaling pathway. The proposed approach will be a valuable resource in the identification and characterizing of signaling networks from phosphoproteomics data.

## Author Summary

A key goal in cell signaling studies is to identify the set of kinases that underlie key signaling events. Mass spectrometry-based technologies have emerged as a powerful tool to profile proteome-wide phosphorylation events *in vivo* at a single amino acid resolution with high precision. However, development of algorithms to analyze and identify signaling events from high-throughput phosphoproteomics data is still in its infancy. Here we propose a knowledge-based CLUster Evaluation (CLUE) approach for identifying key signaling cascades from time-series phosphoproteomics data. Our approach utilizes known kinase-substrate annotations from curated phosphoproteomics databases to first determine the optimal clustering of the phosphorylation sites and then identify enriched kinase(s). We apply CLUE on time-series phosphoproteomics datasets and identify key kinases associated with human embryonic stem cell differentiation and insulin signaling pathway.

## Introduction

Cell signaling controls various aspects of basic cellular processes including homeostasis, proliferation, survival, and cell fate decisions, and defects in mechanisms underlying these processes are associated with a wide range of diseases [1–3]. Protein post-translational modifications (PTMs), which can activate or inhibit protein function/activity, have emerged as key regulators of various signaling pathways [4]. Protein phosphorylation is a common type of PTM that increases the functional diversity of the proteome by altering target proteins between active and inactive forms for signal transduction and integration [5]. It is characterized by the addition of a phosphate group by a protein kinase to a serine, threonine, or tyrosine residue on a substrate protein [6]. Traditionally, protein phosphorylation has been studied largely using *in vitro* assays and, more recently, protein chip arrays [7]. However, kinase activities are often less specific *in vitro* compared to *in vivo* [8], and, as a result, *in vitro* analyses often result in a large number of false discoveries. Recent advances in mass spectrometry (MS)-based technologies [9,10] make it possible to profile proteome-wide phosphorylation events *in vivo* for investigating signal transduction cascades [11], understanding complex diseases [12–14], and develop strategies for therapeuitc intervention [15,16]. With isotopic/isobaric labelling techniques and increasingly label-free approach, proteome-wide phosphorylation events can now be identified and quantified at a single amino acid resolution with high precision [17,18].

A key goal in a phosphoproteomics study is to identify the set of kinases and their corresponding substrates that underlie key signaling events [19]. Much progress has been made on developing computational tools to predict substrates of a given kinase using consensus sequence recognition motif [20,21] and incorporating additional information such as protein structure [22] and colocalization [23]. Conversely, computational approaches have been proposed to identify kinases based on substrate recognition motifs and differentially phosphorylated substrates [16,24–27]. It is estimated that there are over 500 kinases in human cells [28]. Most kinases phosphorylate not only many proteins but also many sites on the same protein. By analyzing phosphorylation sites (substrates) proteome-wide over a course of time, the dynamics of signaling cascades can be elucidated [29]. Since many substrates of a given kinase have similar temporal kinetics, clustering phosphorylation sites into distinctive clusters can facilitate identification of their respective kinases [8,30–33]. To identify the kinases that underlie key signaling cascades, clustering algorithms such as *k*-means clustering and its variant fuzzy *c*-means clustering are frequently utilized to partition the phosphorylation sites into clusters with distinctive temporal profiles from which the corresponding kinases and their activity could be inferred [8,30–33]. Fuzzy *c*-means clustering is an extension of the classic *k*-means clustering that allows a phosphorylation site to be assigned to multiple clusters with probabilistic “membership” scores [34]. While *k*-means clustering-based algorithms are computationally efficient and provide an intuitive separation and summarization of the temporal profiles [35,36], their performance can be strongly influenced by the user-selection of the parameter *k*, which dictates the partitioning of the data into exactly *k* clusters. Thus, estimation of *k* becomes critical to generating biologically meaningful clusters. An underestimation of *k* will force unrelated phosphorylation sites to be assigned to the same cluster whereas an overestimation will split related phosphorylation sites across two or more clusters [37], hence confounding downstream analyses.

Numerous methods and metrics have been proposed over the years to estimate the optimal choice of *k* for *k*-means clustering-based algorithms. Popular approaches include internal indices such as Dunn index [38] and Connectivity [39], stability indices such as average proportion of non-overlap (APN), average distance (AD), average distance between means (ADM) [40], and the figure of merit (FOM) [41], and biological indices that measure biological homogeneity (BHI) or biological stability (BSI) [42]. However, none of these approaches assess the information content of resulting clusters using a formal hypothesis testing framework, nor are they specifically designed for analyzing phosphoproteomics data. Here we propose a knowledge-based CLUster Evaluation (CLUE) approach for determining the most informative partitioning of a given temporal phosphoproteomics data using a hypothesis testing approach. Our approach utilizes known kinase-substrate annotations from curated phosphoproteomics databases to first estimate the optimal number of clusters within a dataset and then identifies the enriched kinase(s) associated with each cluster. Using simulation studies, we show that CLUE outperforms several alternative approaches in identifying the optimal number of clusters. In addition, we apply CLUE on time-series phosphoproteomics datasets [12,43] and identify key kinases associated with human embryonic stem (hES) cell differentiation and insulin signaling in 3T3-L1 adipocytes.

## Results

### Overview of CLUE approach

**Fig 1:**
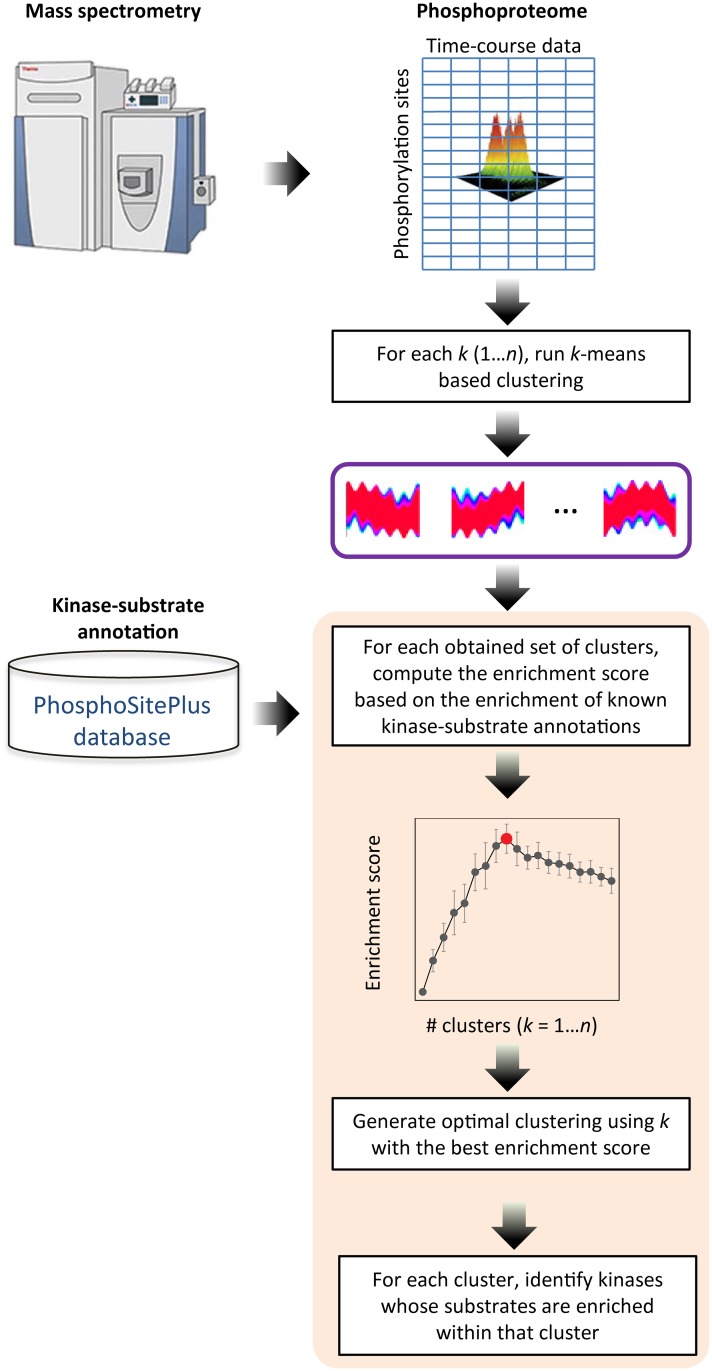
Schematic overview of CLUE. The level of phosphorylation for each phosphorylation sites in the proteome are quantified in time-course by mass spectrometry. First, time-course profiles of phosphorylation sites are partitioned into clusters using a *k*-means clustering-based algorithm for a range of values for *k*. Next, the clustering result, for each *k*, is evaluated based on the correct clustering of known substrates of kinases, as annotated in the PhosphoSitePlus database [53], and an enrichment score is computed. The clustering with the highest enrichment score is reported as the optimal clustering along with kinases whose substrates are enriched within each cluster.

Identification of key kinases that control the activation and inhibition of cell signaling is a critical step for characterizing signaling cascades in time-course phosphoproteomics studies. Since many substrates (phosphorylation sites) of a given kinase are may have similar temporal profiles, partitioning phosphorylation sites from a proteome-wide time-series study into informative clusters, each with a distinctive temporal profile, becomes vital toward identification of kinases that could explain the observed phosphoproteome. We developed a knowledge-based CLUster Evaluation (CLUE) framework that uses existing knowledge, known kinase-substrate annotations from curated phosphoproteomics databases, to guide the generation of biologically meaningful clusters. A schematic overview of CLUE is presented in (Fig 1). CLUE provides a framework to assess the most informative partitioning of a given temporal phosphoproteomics data. Specifically, CLUE estimates the optimal *k* for clustering data using *k*-means clustering-based algorithms (see Materials and Methods for details).

### CLUE's performance over alternative approaches

**Fig 2:**
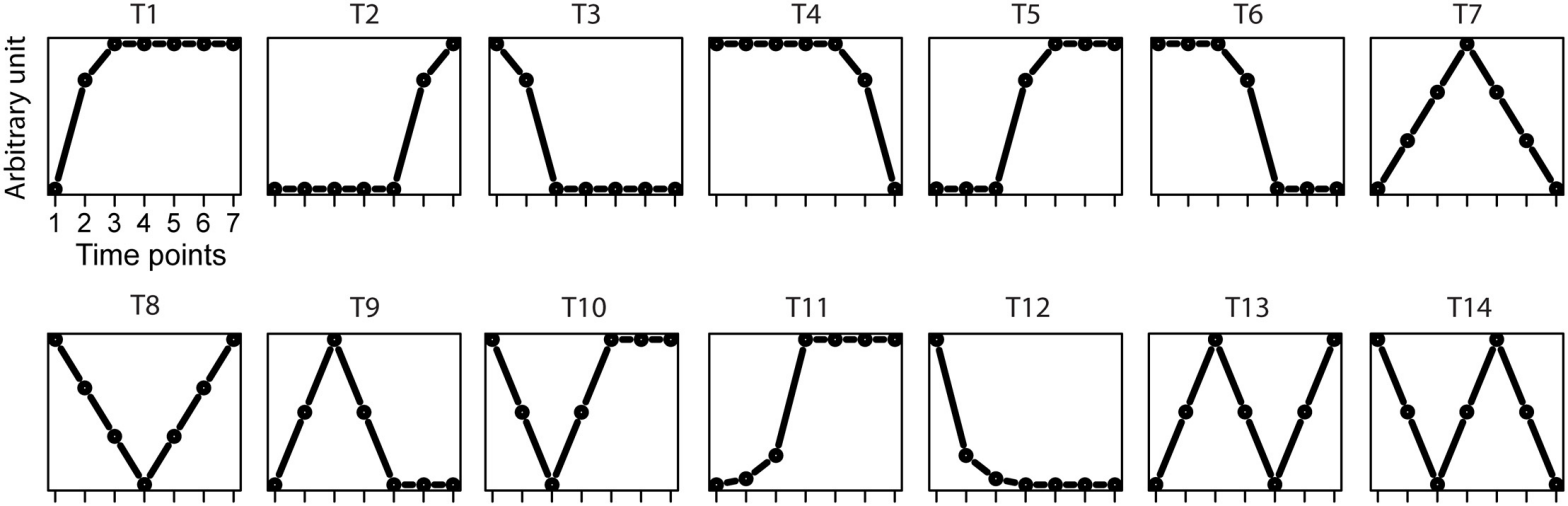
Temporal profile templates used in simulation studies. Fourteen temporal profiles templates, each with seven time points and a unique time-course pattern, were defined for generating simulation datasets. For each time point, a random variable with a defined Gaussian distribution is used to generating the temporal profile for the simulation datasets.

To assess CLUE's ability to partition data into meaningful clusters and to assess CLUE's performance against alternative approaches for estimating *k*, we conducted studies using simulated phosphoproteomics data (see Materials and Methods for details). We generated scenarios where the data were simulated to have varying number of clusters. In each case, the clusters were generated based on a set of randomly selected temporal profile templates (Fig 2), each representative of a phosphorylation activity profile over seven time points. The goal was to assess how well each method performs in recovering the true number of clusters. We compared CLUE with eight popular approaches including those that use internal indices such as Dunn index [38] and Connectivity [39], stability indices such as average proportion of non-overlap (APN), average distance (AD), average distance between means (ADM) [40], and the figure of merit (FOM) [41], and biological indices that measure biological homogeneity (BHI) or biological stability (BSI) [42].

Every method we tested computes an objective score for each *k* and reports the *k* with the best score. To facilitate a fair comparison of methods, we transformed the objective scores from each method into the range [0, 1] by using Min-Max normalization. After the normalization, the scores from methods that seek to minimize the objective function were further transformed into 1 minus the normalized Min-Max scores. First, we compared the performances of CLUE and other commonly used approaches including Dunn index, Connectivity, APN, AD, ADM, FOM, BHI, and BSI in estimating the optimal number of clusters for each of the scenarios with simulated data. In all cases, the fuzzy *c*-means clustering, an extension of the classic *k*-means clustering, was used to partition the data, and the results were largely the same even when *k*-means clustering was used.

**Fig 3:**
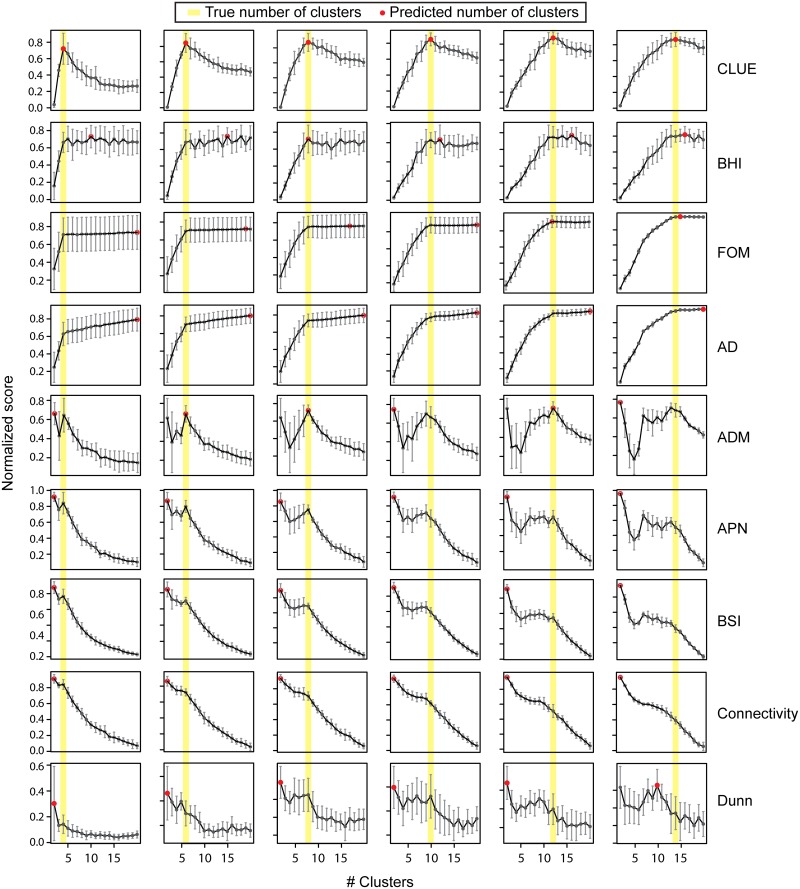
Comparison of CLUE with alternative approaches. Raw scores, representing the quality of clustering result for each *k*, for each method were normalized to be between 0 and 1 (y-axis). The higher the score, the more informative the resulting clustering is. The methods were evaluated based on how accurately they can recover the true number of clusters within a simulated dataset. The yellow line represents the true number of clusters in the simulated dataset, and the red dot denotes the predicted number of clusters in each case.

Results from our simulation studies (Fig 3) reveal that in all cases, CLUE was able to accurately identify the true number of clusters in the simulated datasets whereas other methods were not as accurate. Importantly, the simulation studies also revealed some common biases with some of the methods tested. In particular, BHI, FOM, and AD have a tendency to overestimate the optimal number of clusters. In other words, while these methods are able to capture the lower bound on the optimal number of clusters, they fail to provide a reasonable upper bound. On the other hand, ADM, APN, BSI, Connectivity, and Dunn index appear to suffer from local optima and thus have a tendency to underestimate the optimal number clusters. In all cases, APN, BSI, and Connectivity reported the optimal number of clusters as 2, severely underestimating the true number of clusters. Although ADM appears to somewhat overcome the bias, it still suffers from local optima. While it is arguable that by observing the pivotal point in the reported scores, several of these methods may help in determining the optimal number of clusters when the true number of cluster is small, such a pivotal point may be less apparent when the number of true clusters is rather large, as one would expect in a high-throughput dataset. Although CLUE, BHI, and BSI utilize known kinase-substrate annotations in aiding their clustering evaluation process, their performances vary significantly perhaps due to how they utilize this information. CLUE's ability to make reasonably accurate predictions on the optimal number of clusters is attributable to it taking advantage of known information and using it to assess and penalize under/over clustering as it attempts to estimate the optimal number of clusters (see Materials and Methods). Similar results were obtained when the classic *k*-means clustering was used instead of the fuzzy *c*-means clustering (S1 Fig), indicating that CLUE's performance is not dependent on the type of *k*-means clustering-based algorithm. Together, these results highlight the advantages of using known kinase-substrate annotations in aiding optimal clustering of phosphoproteomics data.

### CLUE's performance as a function of completeness/accuracy of known kinase-substrate annotations

**Fig 4:**
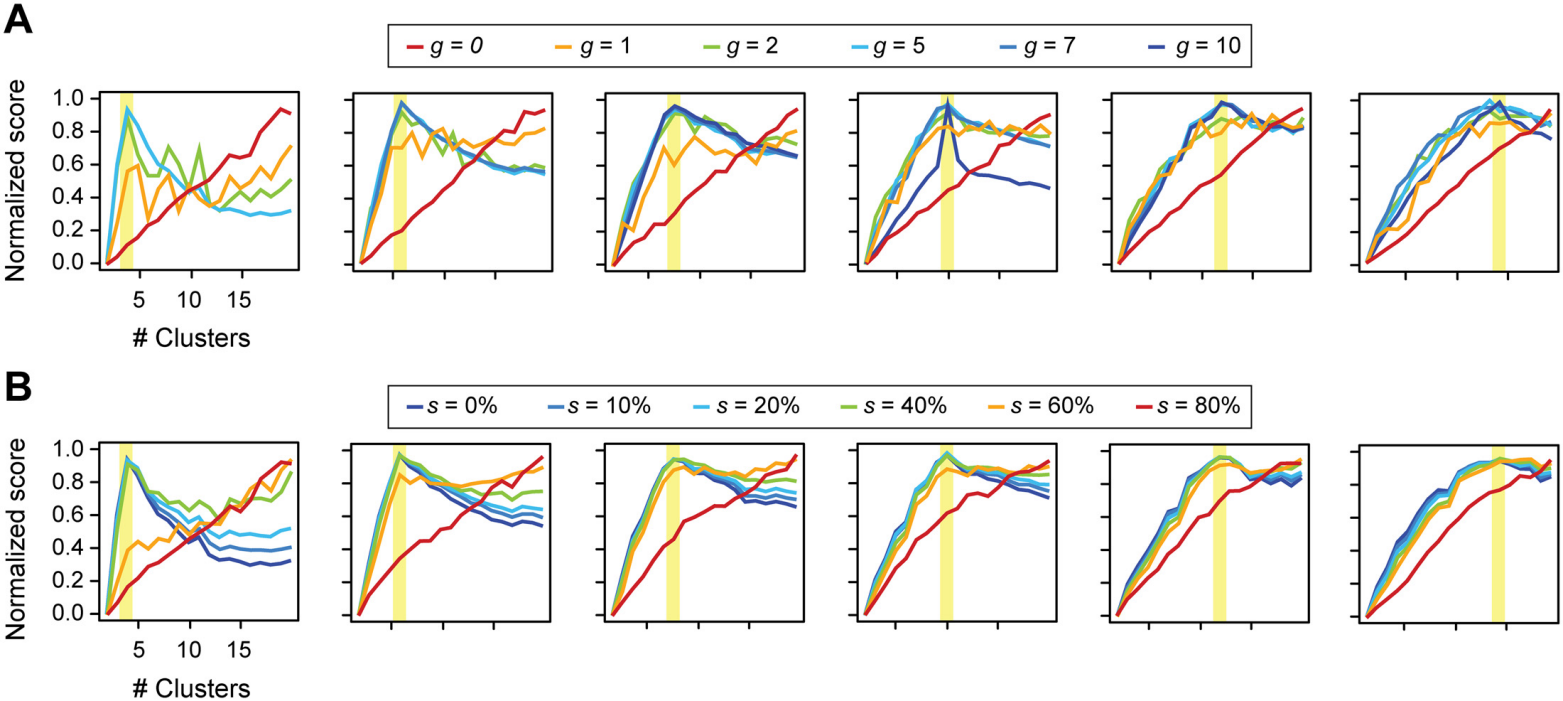
The effects of completeness/accuracy of known kinase-substrate annotations on CLUE's performance. CLUE's performance as a function of number of kinases annotated to have substrates in *g* out of the *k* clusters. The panels (from left to right) show six scenarios with true number of true simulated clusters highlighted in yellow. The scenario *g* = 0 resembles the situation when no existing knowledge is available for use by CLUE. CLUE's ability to accurately predict the true number of clusters improves dramatically as *g* increases. CLUE's performance as a function of percentage of incorrect kinase-substrate annotations (noise). We set *g* = 5 for testing different levels of noise (denoted as *s*). The panels (from left to right) show six scenarios with true number of true simulated clusters highlighted in yellow.

Next, to assess how important the completeness of the known kinase-substrate annotations is in determining CLUE's performance, we simulated data such that only those kinases that had annotations for substrates in *g* out of the *k* clusters were considered. The goal of this simulation study was to determine how much known information is sufficient to help guide optimal clustering of the data. The scenario when *g* = 0 resembles the situation when no existing knowledge is available for use by CLUE. For a method that was designed to rely heavily on existing knowledge to aid clustering, CLUE, as expected, is unable to correctly predict the true number of clusters in the simulated data when *g* = 0 (Fig 4A). However, as *g* is set to higher values, CLUE's ability to accurately predict the true number of clusters improves dramatically.

Having established how valuable existing knowledge is in aiding correct clustering of high-throughput phosphoproteomics data, we next sought to assess the extent to which incorrect annotations (noise) may influence CLUE's performance. To this end, we simulated different levels of noise by requiring 10%, 20%, 40%, 60% or 80% of the substrates to have incorrect kinase assignments, similar to what one might encounter in real-world. As one would expect, CLUE performed poorly when the noise was set at 80% (Fig 4B). However, CLUE was able to consistently recover the true number of clusters even when a substantial percentage, up to ~40%, of the annotation is incorrect. Overall, these simulation results demonstrate that CLUE is robust and powerful in estimating the true number of clusters based on simulated phosphoproteomics data.

### CLUE's performance as a function of data noise and number of time points

Given that later time points post stimulus in phosphoproteomics studies capture non-functional phosphorylation [44], we sought to assess CLUE’s performance as a function of “noisy” data wherein last one or two time points were simulated to be random noise, reflecting non-functional phosphorylation. As expected, we observed a noticable drop in CLUE’s performance with increasingly more time points affected by noise (S2 Fig). This observation highlights the importance of time point selection in phosphoproteomics experimental design. We also assessed CLUE’s performance as a function of the number of profiled time points. In theory, the more the number of time points, the more the chances of capturing the subtle differences in the temporal kinetics, and thus the more the number of clusters one may infer. To test this, we varied the number of time points used for representing temporal patterns in the simulation studies. Specifically, we compared results based on data from all seven time points against those from four (1, 3, 5, 7) or three (1, 4, 7) time points. Although using data from just four time points correctly predicted the number of true clusters, the levels of uncertainty was noticeably higher (error bars in S2 Fig, middle panel). Using data from fewer (three) time points leads to underestimation of the true number of simulated clusters (S2 Fig, right panel). Thus, we conclude that the number of time points required for dissecting various kinases depends on the profiled signaling processes. If the signaling processes have complex temporal features, fewer than sufficient number of time points may not provide the necessary resolution to distinguish them from each other and CLUE will likely group them into a single cluster.

### Using CLUE to Identify key signaling events from phosphoproteomics data

To demonstrate how valuable CLUE would be in identifying key signaling events from high-throughput phosphoproteomics data, we applied CLUE on two previously published SILAC-based temporal phosphoproteomics datasets on differentiating human embryonic stem (hES) cells (five time points) [43] and insulin activation in mouse 3T3-L1 adipocytes (nine time points) [12].

#### Human embryonic stem cell differentiation

**Fig 5:**
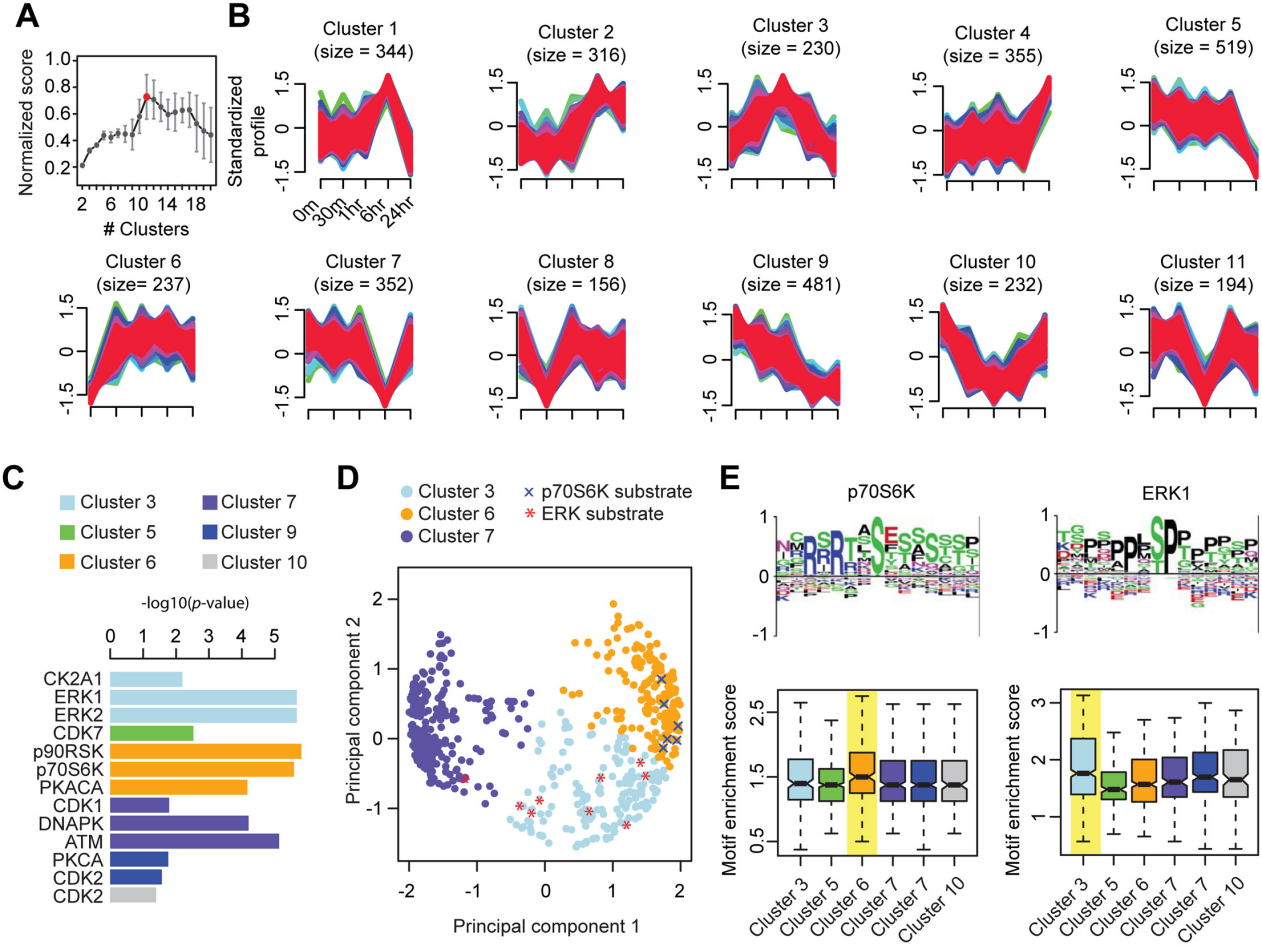
Optimal clustering and analysis of hES cell phosphoproteomics data. CLUE's estimation of number of clusters. The number of clusters evaluated ranges from 2 to 20 and the optimal number of clusters, as estimated by CLUE, is highlighted in red. Visual representation of temporal profiles of phosphorylation sites within each cluster. Membership scores of all phosphorylation sites within a cluster is used to create color gradient from green to red correspond to lower to higher clustering confidence. Size: number of phosphorylation sites that have membership in that cluster. Bar plot showing kinases whose substrates are enriched within each cluster (*p*-value < 0.05; Fisher’s exact test). Principal component analysis of the temporal profile of phosphorylation sites within clusters 3, 6, and 7. Known substrates of p70S6K and ERK kinases are highlighted as x and *, respectively. Motif enrichment analysis. Phosphorylation sites from each cluster are scored against the PSSMs of p70S6K and ERK1, respectively. The cluster with the highest motif enrichment scores (median) are highlighted in yellow.

CLUE estimated the optimal number of clusters in hES cell differentiation dataset to be 11 (Fig 5A and S1 Table). The temporal profiles of substrates within clusters generated using the *c*-means clustering with *c* = 11 are shown in Fig 5B. Evaluation of substrates within each cluster against known kinase-substrate annotations revealed enrichment of substrates known to be phosphorylated by specific kinases (Fig 5C, 5D and 5E and S2 Table). Notably, substrates of kinases p90RSK, p70S6K, and PKACA (catalytic subunit of cAMP-dependent protein kinase alpha (PKA)) from the AGC subfamily [45] are enriched in a single cluster (cluster 6). The temporal profile of cluster 6 shows acute activation of this pathway within 30 minutes of hES cell differentiation initiation (Fig 5B). The enrichment of p90RSK (*p* = 1.5 x 10–6), p70S6K (*p* = 2.6 x 10–6), and PKACA (*p* = 6.8 x 10–5) substrates within a cluster suggests a role for AGS subfamily of kinases in the signaling cascades critical for the hES cells to exit from their self-renewing pluripotent state. Indeed, consistent with the fast activation of p70S6K substrates during hES cell differentiation (Fig 5B), a previous study of mTOR/p70S6K pathway in hES cells showed that differentiation can be induced by simply overexpressing constitutively active p70S6K [46]. In contrast, the substrates of CDK2 are found to be enriched in cluster 9 with a decreasing activity through profiled time points. Together, these results are consistent with findings from the original study which reported an increased activity of PKA and a decreased activity of CDK2 [43]. Another key kinase known to play a role in embryonic stem cell signaling is the extracellular signal-regulated kinase (ERK) [47,48]. Consistent with ERK's role in embryonic stem cell differentiation, we find an enrichment of ERK substrates (*p* = 2.1 x 10–6) among those in cluster 3 (Fig 5C, 5D and 5E), suggesting an important role for ERK signaling in hES cell differentiation.

#### Insulin activation

**Fig 6:**
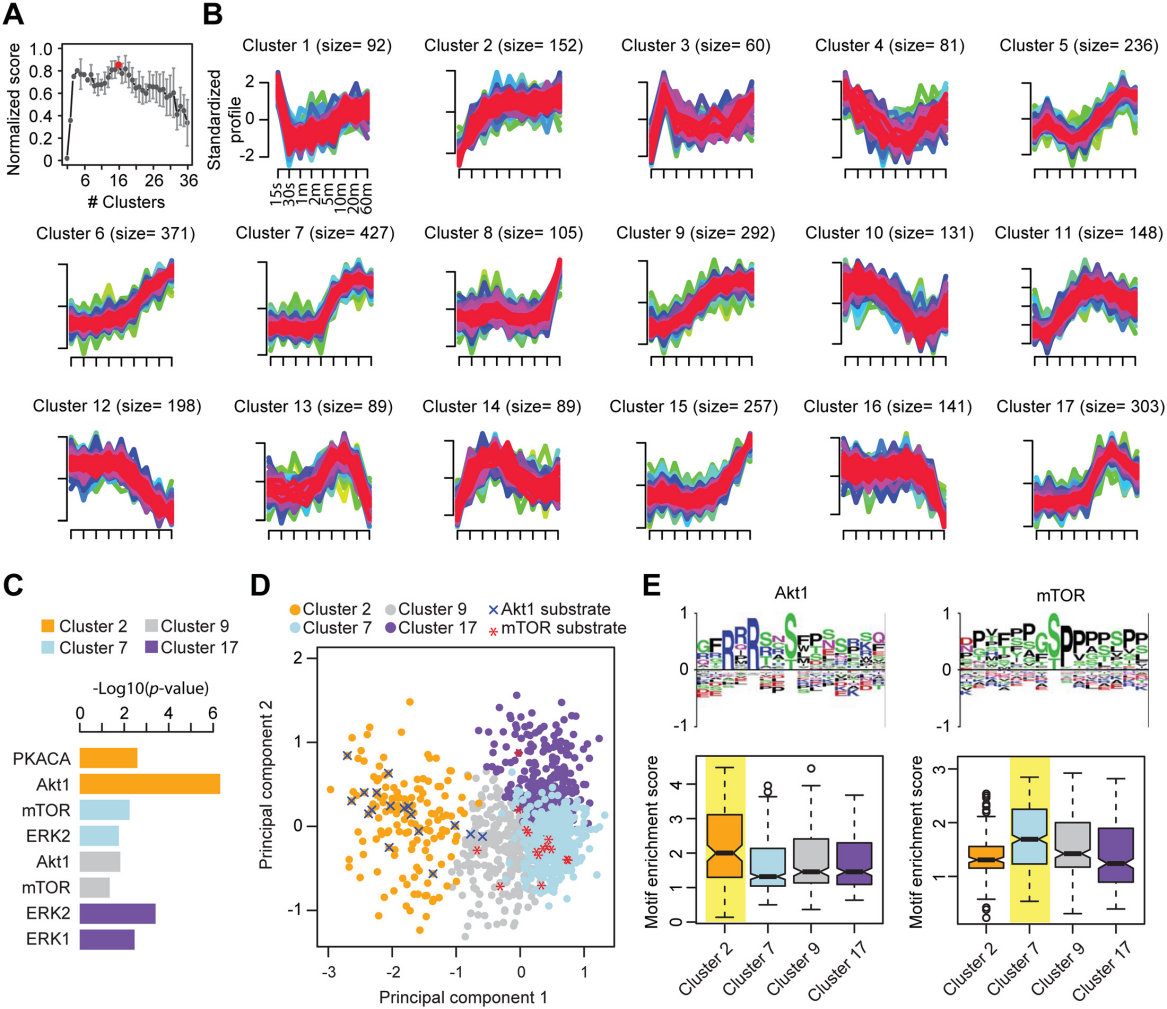
Optimal clustering and analysis of adipocytes phosphoproteomics data. CLUE's estimation of number of clusters. The number of clusters evaluated ranges from 2 to 36 and the optimal number of clusters, as estimated by CLUE, is highlighted in red. Visual representation of temporal profiles of phosphorylation sites within each cluster. Membership scores of all phosphorylation sites within a cluster is used to create color gradient from green to red correspond to lower to higher clustering confidence. Size: number of phosphorylation sites that have membership in that cluster. Bar plot showing kinases whose substrates are enriched within each cluster (*p*-value < 0.05; Fisher’s exact test). Principal component analysis of the temporal profile of phosphorylation sites within clusters 2, 7, 9 and 17. Known substrates of Akt1 and mTOR kinases are highlighted in x and *, r*e*spectively. Motif enrichment analysis. Phosphorylation sites from each cluster are scored against the PSSMs of Akt1 and mTOR, respectively. The cluster with the highest motif enrichment scores (median) are highlighted in yellow.

For the insulin stimulated adipocyte dataset, CLUE estimated the optimal number of clusters to be 17 (Fig 6A and S1 Table). Fig 6B shows the temporal profiles of substrates within clusters generated using the *c*-means clustering *c* = 17. We found substrates of many kinases known to respond to insulin activation enriched in our clustering result (Fig 6C, 6D and 6E and S2 Table). Specifically, cluster 2 is enriched for a group of fast responding substrates upon insulin stimulation. The kinases that are found to be highly enriched in this cluster are Akt1 (*p* = 4.8 x 10–7) and PKACa (*p* = 2.5 x 10–3). Interestingly, phosphorylation sites in cluster 7 are enriched for mTOR substrates (*p* = 5.7 x 10–3), which is known to act downstream of Akt1 in the insulin pathway [12]. As one would expect, the temporal profiles of sites in cluster 7 (mTOR) exhibits relatively delayed activation compared to sites within cluster 2 (Akt1). While it is clear that most of the known Akt1 and mTOR substrates are partitioned into clusters 2 and 7, respectively (Fig 6D), a few known substrates of Akt1 and mTOR are grouped together in cluster 9, with a temporal profile suggesting prolonged activation (Fig 6B). We also find an enrichment for ERK substrates in cluster 17 (*p*<3.5 x 10–5) (Fig 6C). ERK pathway is known to play an important role in insulin signaling [49] and is known to intersect with Akt1/mTOR pathway to co-regulate downstream functions [50]. Our analyses revealed that while Akt1 substrates respond much faster to insulin stimulation than mTOR substrates which are consistent with the results reported by the orginal study [12].

We compared CLUE's performance in recovering known kinases associated with hES cell differentiation and insulin activation with those by other approaches and found that CLUE can reliably recover kinases that underlie these two processes (Table 1). Taken together, these results demonstrate the usefulness of CLUE in facilitating the discovery of key signaling events from temporal phosphoproteomics data by generating biologically meaningful clusters.

**Table 1:** Comparison of CLUE with alternative approaches on the two phosphoproteomics datasets.

	hES cell differentiation					Insulin activation				
Method	Estimated # cluster	Enrichment based on Fisher's Exact Test				Estimated # cluster	Enrichment based on Fisher's Exact Test			
		P70S6K	P90RSK	PKACA	ERK1/2		Akt1	PKACA	mTOR	ERK1/2
CLUE	11	2.6x10-6	1.5x10-6	6.8x10-5	2.1x10-6	17	4.8x10-7	2.5x10-3	5.7x10-3	3.5x10-5
Dunn	7	1.7x10-6	3.4x10-4	1.3x10-4	NS	4	2.7x10-3	1.3x10-3	ns	1.1x10-6
BHI	22	2.2x10-7	5.3x10-4	ns	4.1x10-5	4	2.7x10-3	1.3x10-3	ns	1.1x10-6
connectivity	2	2.0x10-2	ns	ns	ns	2	ns	ns	3.1x10-2	ns
BSI	2	2.0x10-2	ns	ns	ns	2	ns	ns	3.1x10-2	ns
APN	2	2.0x10-2	ns	ns	ns	2	ns	ns	3.1x10-2	ns
ADM	2	2.0x10-2	ns	ns	ns	2	ns	ns	3.1x10-2	ns
AD	>30	-	-	-	-	>30	-	-	-	-
FOM	>30	-	-	-	-	>30	-	-	-	-

ns, not significant;-, not applicable

## Discussion

Identification of key kinases that control activation and inhibition of specific signaling events is critical for characterizing signaling networks. In this study, we described a knowledge-based CLUster Evaluation (CLUE) approach that enables identification of key signaling events from temporal phosphoproteomics data by utilizing known kinase-substrate annotations. Our simulation studies show that CLUE outperforms many alternative methods in recovering the underlying clusters from temporal datasets. To test how CLUE can be utilized for real-world applications, we analyzed temporal phosphoproteomics datasets generated from hES cell differentiation and insulin activation of adipocytes. The understanding of self-renewal and differentiation of hES cells is a subject of major scientific interest due to its applications in cancer treatment and regenerated medicine [51]. It is widely acknowledged that signaling pathways play critical roles in maintaining the pluripotent state of ES cells [52] and therefore, the identification of kinases that are involved in hES cell self-renewal and differentiation is of great importance. Similarly, the insulin signaling pathway plays a key role in regulating and maintaining the physiology of the adipocytes. Therefore, the characterization of the kinases that are the key components in insulin signaling allows potential clinical application to be targeted at different pathway levels. Using CLUE, we were able to identify and characterize several known and novel kinases that are key regulators in hES cell differentiation and insulin signaling. Furthermore, CLUE can also be used to discover novel substrates for active kinases of interest. For instance, in our analyses of the insulin activation data, many known Akt substrates (AS160 Ser595, PFKFB2 Ser469, and BAD Ser136) and mTOR substrates (FRAP Ser2481 and IRS1 Ser632) that have not yet been annotated in PhosphoSitePlus are ranked highly based on the membership score of *c*-means clustering (S1 Table). Thus, not only does CLUE help in the identification of key kinases but also may facilitate identification of novel substrates of kinases.

It is conceivable for a phosphatase to coordinately dephosphorylate a subset of substrates of a given kinase, in which case a subset of substrates of that kinase is expected to exhibit a similar temporal profile and thus clustered together in our analysis. Moreover, increases as well as decreases in substrate phosphorylation levels of a given kinase could be due to elevated (reduced, resp.) kinase activity and/or reduced (increased, resp.) levels of corresponding phosphatase. Either way, even in the absence of phosphatase-substrate information, as long as substrates that belong to a key signaling cascade exhibit similar temporal profile (increasing/decreasing), CLUE will infer them to belong to a cluster and identify putative kinases associated with the cluster. Depending on whether the phosphorylation levels of the substrates within a cluster over the time-course are up/down, one can infer whether that signaling pathway is activated or inactivated. For example, in our analysis of the hES data (Fig 5), we identify enrichment of substrates for ERK (cluster 3) and p70S6K (cluster 6). Based on the temporal profiles, it is evident that ERK signaling is inactivated as hES cells differentiate (beginning 1hr time point), which is consistent with an essential role for ERK signaling in the maintenance of the pluripotent state in hES cells by blocking neuronal, trophectoderm and primitive endoderm differentiation [47]. In contrast, substrates predicted to be that of p70S6K are activated during hES cell differentiation, consistent with the fact that activation of p70S6K alone is sufficient to induce hES differentiation [46]. Thus, CLUE is applicable to analyze both increasing and decreasing phosphorylation profiles and will be useful even when phosphatase-substrate information is unavailable.

Other factors such as protein translation rate, degradation rate, and cell cycle progression may affect phosphorylation especially at later time points, and diverse substrates of a given kinase may be modulated with different kinetics. To address these confounding factors, phosphorylation sites and time points may be pre-filtered to select those that are biologically most relevant for capturing a given kinase’s activity when such prior knowledge is available.

Our simulation studies reveal that CLUE's performance is dependent on the accuracy of the annotations (prior knowledge) that is employed to aid the clustering process. Although CLUE can tolerate reasonable amount of noise/inaccuracies (up to ~40%), using annotations from a high quality source/database is essential for accurate and biologically meaningful clustering of the data. It is worth noting that CLUE's performance is not biased towards larger kinase-substrate annotation groups as Fisher’s Exact test used to test for kinase enrichment is robust to size differences in kinase-substrate annotations.

Although we formulated CLUE for analyzing phosphoproteomics data, the general framework of CLUE can also be used to analyse temporal transcriptomics data toward identification of transcription networks and cascades. This can be accomplished by using gene set annotations, as defined by various gene ontology-like databases, or transcription factor-target gene annotations in place of kinase-substrate annotations. While CLUE is designed to perform optimally with *k*-means clustering-based algorithms, in theory, it can be coupled with other clustering algorithms such as SOM where the cluster enrichment can be evaluated.

## Materials and Methods

### Kinase-substrate annotation

Kinase-substrate annotations were compiled from the PhosphoSitePlus database, a curated database of protein post-translational modifications (PTMs) including phosphorylation [53]. We compiled mouse-specific and human-specific kinase-substrate annotations and assigned to each kinase its phosphorylation substrates from mouse and human, respectively, based on “KINASE”, “SUBSTRATE”, and “SUB_ORG” columns of the database. The official gene symbols and the phosphorylated residues (amino acids) were concatenated together to create unique identifiers for each phosphorylation site. Phosphorylation sites assigned to multiple kinases (in PhosphoSitePlus) are classified to multiple kinases in the enrichment analysis. In total, we extracted 206 kinases and 9830 kinase-substrate interactions for human, and 235 kinases and 17532 kinase-substrate interactions for mouse.

### Knowledge-based CLUster evaluation (CLUE) framework

CLUE relies on annotated kinase-substrate relationships to estimate the optimal *k* for clustering phosphoproteomics data using *k*-means clustering-based algorithms (Fig 1). Given a clustering output from a *k*-means clustering-based algorithm that partitions the data into exactly *k* clusters, let *i* = 1…*k* be the *i* ^*th*^ cluster. Let *m* be the number of kinases annotated in the PhosphositePlus database for the species of interest and *j* = 1…*m* be the *j* ^*th*^ kinase. Let *a*_*ij*_ denote the number of phosphorylation sites regulated by kinase *j* that are included in cluster *i*, *b*_*ij*_ denote the number of phosphorylation sites regulated by kinase *j* that are not present in cluster *i*, *c*_*ij*_ denote the number of phosphorylation sites in cluster *i* that are not regulated by kinase *j*, and *d*_*ij*_ denote the number of phosphorylation sites that are neither included in cluster *i* nor regulated by *j*. Let us define *θ* as odds-ratio such that *θ* = (*a*_*ij*_ / *b*_*ij*_) / (*c*_*ij*_ / *d*_*ij*_), and under Fisher’s exact test, we can test for the significance of enrichment of *j*'s substrates in cluster *i* under the null hypothesis that the substrates of *j* are not over-represented in cluster *i* (i.e. *H*_0_:*θ* = 1) and the alternative hypothesis that the substrates of *j* are over-represented in *i* (i.e. *H*_1_:*θ* > 1). For a given set of values *a*_*ij*_, …, *d*_*ij*_, the enrichment can best tested as follows:

$$prob_{ij}=\frac{\left(\begin{array}{c} a_{ij}+b_{ij}\\ a_{ij} \end{array}\right)\left(\begin{array}{c} c_{ij}+d_{ij}\\ c_{ij} \end{array}\right)}{\left(\begin{array}{c} a_{ij}+b_{ij}+c_{ij}+d_{ij}\\ a_{ij}+c_{ij} \end{array}\right)}$$

and the *p*-value for the test of significance (i.e. *p*_*ij*_) is obtained by summing the *prob*_*ij*_ values over all combinations of *a*_*ij*_, …, *d*_*ij*_ that return odds-ratio values at least as large as the observed values.

By applying the above test for all *m* kinases against a given cluster *i*, the significance of the information content of cluster *i* is determined as follows:

$$p\left(cluster_{i}\right)=\underset{}{{\text{min}}_{j=1\ldots m}}\left(p_{ij}\right).$$

Then, the *p*-values for all *k* clusters are combined using Fisher’s combined probability test:

$$P_{k}=\text{P}\left(\chi_{d}^{2}>-2\overset{k}{\underset{i=1}{\sum^{\text{​}}}}\text{log}\left(p\left(cluster_{i}\right)\right)\right),$$

where *d* = *2k* denotes the degrees of freedom. Finally, *P*_*k*_ is converted into an enrichment score *E*_*k*_ = -log10(*P*_*k*_), which indicates how informative it is to partition the data into *k* clusters. The higher the enrichment score, the more informative the resulting clustering is. The enrichment score captures both the information content of each individual cluster while also assessing the overall enrichment of the entire partitioning. Intuitively, with an overestimated *k*, phosphorylation sites that are substrates of a kinase might be split across two or more clusters, which will be penalized by Fisher’s exact test for lower information content of resulting clusters. In contrast, underestimation of *k* might group unrelated phosphorylation sites to the same cluster, which will be penalized by Fisher’s combined probability test. By using *k*-means clustering-based algorithm with a range of different *k* values to partition the dataset and assessing the enrichment score for each *k* using CLUE, the optimal *k* for partitioning can be estimated.

### Simulation studies

To compare CLUE's performance with those of other commonly used approaches for estimating *k* for *k*-means clustering-based algorithms, we conducted simulation studies. First, we defined 14 temporal profiles, each with seven time points, representing typical temporal kinetics observed in a time-series study (Fig 2). Next, time course phosphorylation profiles for individual sites (substrates) were simulated by randomly selecting a set of temporal profiles, representing a set of clusters, from the 14 templates and then generating data using the selected temporal profiles with Gaussian noise. Specifically, 500 phosphorylation sites were generated for each temporal profile under a Gaussian distribution with the standard deviation held constant (*σ* = 1). For instance, to simulate a 4-cluster dataset, 4 different temporal profile templates are randomly selected and a total of 2000 phosphorylation sites are generated based on the selected temporal profile templates. In the case of simulating a 14-cluster dataset, all temporal profiles templates are used and a total of 7000 phosphorylation sites are generated. Then, we evaluated CLUE's performance using the *k*-means as well as the fuzzy *c*-means clustering algorithms. For the purposes of testing, we used values for *k* (or *c* in the case of fuzzy *c*-means clustering) ranging from 2 to 20. In practice, this can be specified by the user. Since the *k*-means and the fuzzy *c*-means clustering algorithms randomly initiate centroids, for each *k* (or *c*, respectively), clustering was performed 10 times, each time with a different initialization of centroids in order to obtain an estimation of means. The final result is obtained by averaging the results from each individual runs, and the optimal clustering is determined by finding the maximum enrichment score from the final result.

For simulating the database of annotated kinase-substrate relationships, we generated 100 kinase-substrate groups, each comprising 50 substrates assigned to a kinase. For evaluation purposes, of the 100 groups, *g* groups were generated to each contain phosphorylation sites (substrates) defined to have the same temporal profile. To assess the extent to which incorrect annotations (noise) may influence the performance of CLUE, we set *g* = 5 and simulated different levels of noise by requiring 10%, 20%, 40%, 60%, or 80% of the substrates from each group to have a temporal profile different from that of the rest of substrates in that group. The remaining 95 kinase-substrate groups were generated to contain substrates that were randomly sampled from all phosphorylation sites in the simulated dataset. The resulting simulated kinase-substrate annotations were used for the evaluation of CLUE, BSI and BHI in estimating the optimal number clusters in the simulation experiments.

### Temporal phosphoproteomics datasets

To demonstrate the utility of the proposed approach, we applied it on two previously published SILAC-based temporal phosphoproteomics datasets on (a) human embryonic stem (hES) cells differentiation using phorbol 12-myristate 13-acetate (PMA) treatment [43] and (b) insulin activation in mouse 3T3-L1 adipocytes [12]. The hES cell differentiation data has a total of 14,865 unique phosphopeptides containing 23,522 phosphorylation sites mapping to 4,335 proteins. The phosphopeptides were quantitated over a time-course of five time points during hES cell differentiation (0 min, 30 min, 1 hour, 6 hour, and 24 hour). For clustering analyses, only those phosphorylation sites that have an associated gene product and at least 2-fold change in phosphorylation levels at any time point during differentiation compared to the initial time point (0 min) were considered. This filtering step resulted in 3,416 phosphorylation sites. The insulin activation dataset has a total of 38,901 unique phosphopeptides corresponding to 37,248 phosphorylation sites mapping to 5,705 proteins. The phosphopeptides were quantitated over a time-course of nine time points during insulin treatment of mouse adipocytes (0 sec, 15 sec, 30 sec, 1 min, 2 min, 5 min, 10 min, 20 min, and 1 hour) performed in biological triplicates. For clustering analyses, only those phosphorylation sites that have an associated gene product and are differentially phosphorylated, as determined using a moderated *t*-test implemented in limma R package [54] with a false discovery rate (FDR) of 0.05 as cutoff, were considered. This filtering step resulted in 3,178 phosphorylation sites.

### Motif enrichment analysis

For a given kinase of interest, the amino acid sequences of its substrates annotated in PhosphoSitePlus database is extracted to calculate a position-specific scoring matrix (PSSM) as follows:

$$P_{a,j}=\frac{1}{N}\sum_{i=1}^{N}I\left(x_{i,j}=a\right)$$

where *N* is the number of annotated substrates, *j* is the amino acid position, *a* is the set of characters corresponding to the 20 amino acids, and I is the indicator function. Then, a motif enrichment score is calculated for each phosphorylation site by summing the frequency of occurrence of each amino acid in relation to the PSSM.

### Software implementation

CLUE was implemented as an R package. The source code and documentation are freely available from CRAN (http://cran.r-project.org/web/packages/ClueR/index.html).

## Supporting Information

S1 Fig Simulation results showing CLUE's p*e*rformance using classic *k*-means clustering. The yellow line represents the true number of clusters in the simulated dataset, and the red dot denotes the predicted number of clusters in each case.
(TIF)

S2 Fig Simulation results showing CLUE's performance in relation to data noise and number of time points. The yellow line represents the true number of clusters in the simulated dataset, and the red dot denotes the predicted number of clusters in each case. (A) CLUE’s performance using data from all seven time points (left), data for the last time point simulated as random noise (middle), and data for the last two time points as random noise (right). (B) CLUE’s performance using data from all seven time points (left), data from four (1, 3, 5, 7) time points, and data from three (1, 4, 7) time points.
(TIF)

S1 Table Clustering membership scores for hES cell differentiation and insulin activation datasets. (XLSX)

S2 Table Kinases whose substrate are enriched within identified clusters in hES cell differentiation and insulin activation datasets. (XLSX)
