# Supplementary figures and images for: Knowledge-Based Analysis for Detecting Key Signaling Events from Time-Series Phosphoproteomics Data
Source: PLoS Comput Biol. 2015 Aug 7;11(8):e1004403. doi: 10.1371/journal.pcbi.1004403 (PMC4529189; doi:10.1371/journal.pcbi.1004403)

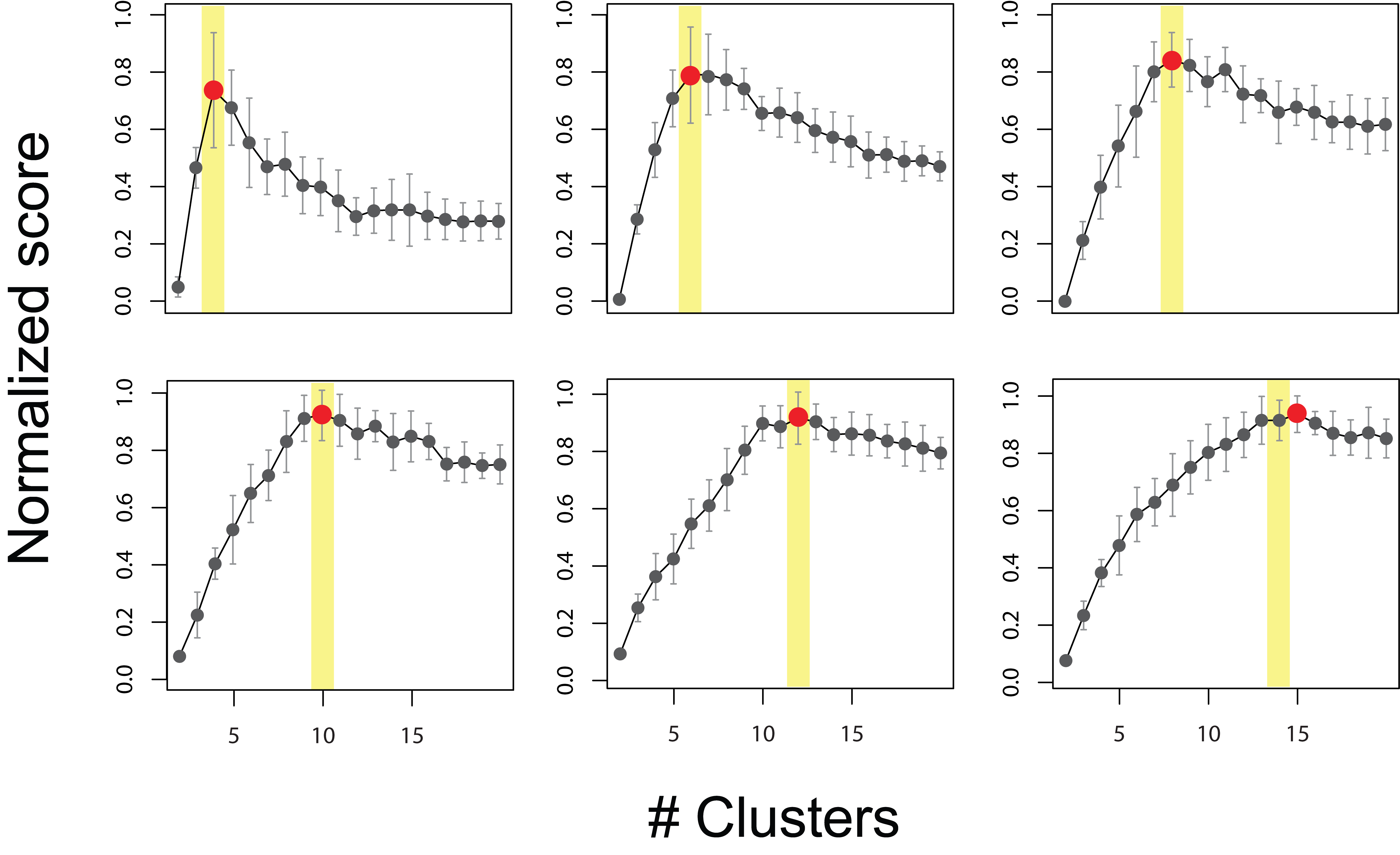

Supplement: S1 Fig — The yellow line represents the true number of clusters in the simulated dataset, and the red dot denotes the predicted number of clusters in each case. (TIF) [file pcbi.1004403.s001.tif]

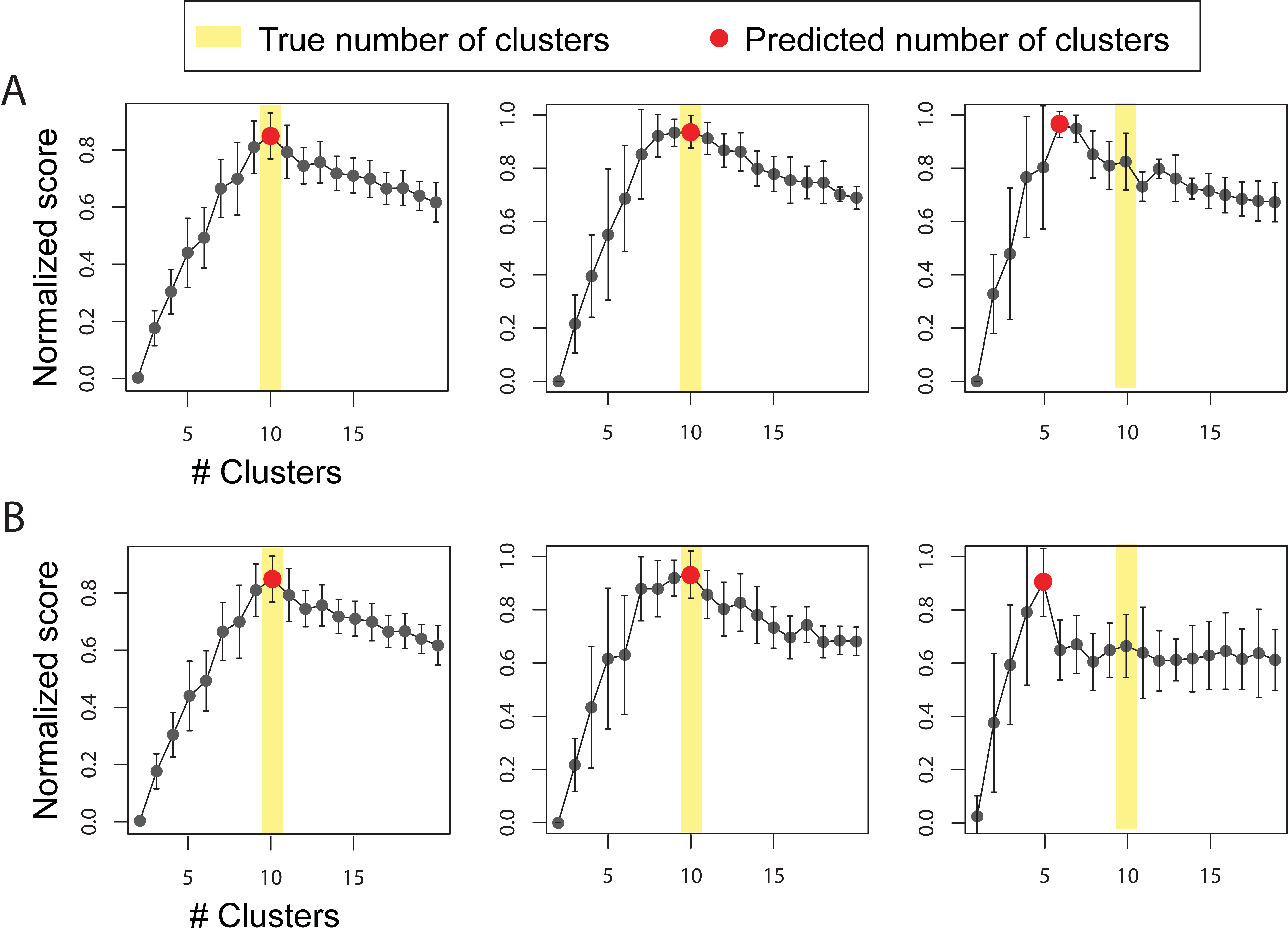

Supplement: S2 Fig — The yellow line represents the true number of clusters in the simulated dataset, and the red dot denotes the predicted number of clusters in each case. (A) CLUE’s performance using data from all seven time points (left), data for the last time point simulated as random noise (middle), and data for the last two time points as random noise (right). (B) CLUE’s performance using data from all seven time points (left), data from four (1, 3, 5, 7) time points, and data from three (1, 4, 7) time points. (TIF) [file pcbi.1004403.s002.tif]
